# Decision Making in Double-Pedicled DIEP and SIEA Abdominal Free Flap Breast Reconstructions: An Algorithmic Approach and Comprehensive Classification

**DOI:** 10.3389/fsurg.2015.00049

**Published:** 2015-10-26

**Authors:** Charles M. Malata, Nicholas G. Rabey

**Affiliations:** ^1^Postgraduate Medical Institute, Faculty of Health Sciences, Anglia Ruskin University, Cambridge and Chelmsford, UK; ^2^Cambridge Breast Unit, Plastic and Reconstructive Surgery Department, Cambridge University Hospitals NHS Foundation Trust, Cambridge, UK; ^3^Department of Plastic and Reconstructive Surgery, Salisbury District Hospital, Salisbury, UK

**Keywords:** Bipedicled free flaps, double-pedicle free flaps, stacked free flaps, breast reconstruction, abdominal free flaps, DIEP and SIEA breast free flaps, intra-flap and extra-flap microvascular anastomoses, rib sparing internal mammary vessel exposure

## Abstract

**Introduction:**

The deep inferior epigastric artery perforator free flap is the gold standard for autologous breast reconstruction. However, using a single vascular pedicle may not yield sufficient tissue in patients with midline scars or insufficient lower abdominal pannus. Double-pedicled free flaps overcome this problem using different vascular arrangements to harvest the entire lower abdominal flap. The literature is, however, sparse regarding technique selection. We therefore reviewed our experience in order to formulate an algorithm and comprehensive classification for this purpose.

**Methods:**

All patients undergoing unilateral double-pedicled abdominal perforator free flap breast reconstruction (AFFBR) by a single surgeon (CMM) over 40 months were reviewed from a prospectively collected database.

**Results:**

Of the 112 consecutive breast free flaps performed, 25 (22%) utilised two vascular pedicles. The mean patient age was 45 years (range = 27–54). All flaps, but one (which used the thoracodorsal system), were anastomosed to the internal mammary vessels using the rib-preservation technique. The surgical duration was 656 min (range = 468–690 min). The median flap weight was 618 g (range = 432–1275 g) and the mastectomy weight was 445 g (range = 220–896 g). All flaps were successful and only three patients requested minor liposuction to reduce and reshape their reconstructed breasts.

**Conclusion:**

Bipedicled free abdominal perforator flaps, employed in a fifth of all our AFFBRs, are a reliable and safe option for unilateral breast reconstruction. They, however, necessitate clear indications to justify the additional technical complexity and surgical duration. Our algorithm and comprehensive classification facilitate technique selection for the anastomotic permutations and successful execution of these operations.

**Levels of evidence:**

Therapeutic level IV.

## Introduction

The free single-pedicled deep inferior epigastric artery perforator (DIEP) flap is the gold standard for autologous breast reconstruction. However, its harvest based on one vascular pedicle may not provide enough viable abdominal tissue in patients who are slim, nulliparous, post massive-weight loss, have midline abdominal scars, or possess minimal abdominal tissue with comparatively large breasts. Double-pedicled abdominal free flaps are designed to overcome this deficit by utilizing most of the lower abdominal tissue for unilateral breast reconstruction. While single-pedicled free TRAM flaps may perfuse more tissue than a single-pedicled DIEP flap they carry a higher donor site morbidity and do not consistently or reliably perfuse Hartrampf Zone 4, which is necessary to harvest a larger flap.

Bipedicled or double-pedicled abdominal perforator flaps are so-named because they are supplied by two anatomically distinct vascular pedicles, thereby increasing the total tissue volume with a Hartrampf Zone I type perfusion (1, 2). Harvesting the entire lower abdomen on two vascular pedicles was pioneered by Arnez and Scamp in 1992 using their bipedicled free TRAM flap design (3). Blondeel and Boecyx, however, were the first to apply this concept to free abdominal perforator flaps in their 1994 description of a DIEP flap (4). Several series have since shown the reliability of bipedicled free DIEP flaps (5–8). However, there are multiple flap configurations and arrangements of the vascular anastomoses (Table 1).

**Table 1:** **Published reports on the abdominal bipedicled flap for breast reconstruction**.

Study	Number of cases	Indications	Flap tissue arrangement	Vascular arrangements	Complications
(4)	1	Midline abdominal scar	Single layer	Primary DIEA to IMV Secondary DIEA to inferior continuity of primary DIEA	Nil
(9)	1	Large contralateral breast, midline abdominal scar	Stacked	Primary DIEA to subscapular artery Secondary DIEA to inferior continuity of primary DIEA	Re-exploration needing vein anastomosis and graft
(6)	16	Previous liposuction (19%), abdominal scars (31%), insufficient tissue volume (50%)	Single layer, folded if required	DIEA/DIEA flaps (43.8%) DIEA/SIEA flaps (43.8%) DIEA/perforator flaps (12.5%) All primary pedicle anastomosis to IMV, secondary pedicle to primary DIEA (81.3%), secondary pedicle to TDAs (18.8%)	• Scar correction (25%) • Donor wound dehiscence (19%) • Fat necrosis (6%) • Lipofilling (6%)
(10)	1	Midline abdominal scar	Stacked	Primary: DIEA to TDA Secondary: SIEA to inferior continuity of DIEA	Nil
(5)	14	Thin abdominal wall (21%), large contralateral breast (C cup or above 57%)	Single layer	DIEA/DIEA (42.9%) DIEA/MS-TRAM (57.1%) Donor vessels • TDA and serratus (21.4%) • TDA and IMV (71.4%) • IMVs anterograde and retrograde (7.1%)	• Cellulitis (7%) • Delayed healing (7%) • Hypertrophic scar (7%) • Local recurrence (7%) • Contralateral mastopexy 14% • Abdominal bulge (7%) • Blood transfusion (21%)
(11)	5	Infraumbilical vertical abdominal scar (100%)	Single layer	Primary DIEA pedicle to IMV. Secondary DIEA pedicle to superior continuity of primary pedicle	Minor fat necrosis (40%)
(7)	96	Previous abdominal scars (31.9%) including midline abdominal scars (25.7%)	Single layer	All used IMVs for primary DIEA pedicle anastomoses. For secondary DIEA pedicle • Superior continuity: 43.7% • Inferior continuity end-to-end: 30.2% • Inferior continuity artery end-to-side: 24.0% • Extra-flap anterograde/retrograde: 2%	• Total Flap Loss – 1.8% • Partial flap loss – 0.9% • Re-exploration – 6.2% • Breast fat necrosis – 10.6% • Abd wound infection: 2.7% • Abdominal bulge (0.9%)
(12)	1	Large contralateral breast	Folded	DIEA/DIEA to IMV extra-flap anterograde/retrograde.	Nil
(13)	55	Insufficient abdominal volume	Folded and stacked	Primary DIEA pedicle (superficial) to IMVs, secondary DIEA pedicle (deep) intra-flap anastomosis to primary pedicle	Hematoma (5.4%)

The blood supply of bipedicled free abdominal flaps may originate from the deep inferior epigastric arteries (DIEAs), superficial inferior epigastric arteries (SIEAs), DIEP perforators, or a combination of these. These options were first classified by Hamdi et al. according to the named vessel and vascular configuration employed (6). Various authors have since utilized similar or different anatomical vascular arrangements (5–8, 14, 15). Hitherto there has, however, been no comprehensive classification. In general the anastomoses of the two pedicles may take the form of an *intraflap* (“in-series”) arrangement as described by Hamdi et al., an *extraflap* (“in-parallel”) arrangement or a *combined intraflap–extraflap* arrangement using the internal mammary vessels (IMVs) in combination with the thoracodorsal or other vessels (9, 10, 16, 17). In the *extraflap* arrangement the two pedicles are independently anastomosed to the IMVs for the primary flap and to the thoracodorsal vessels (TDVs) or retrograde IMVs or other vessels for the secondary flap (6, 10). The *combined intraflap–extraflap* arrangement employs an arterial anastomosis of the secondary flap pedicle to an intraflap site and the venous anastomosis to an extraflap site or vice versa.

Despite the proven efficacy of abdominal bipedicled free flaps (Table 1), the literature is sparse regarding the choice of vessel configuration and flap inset. We therefore deemed it useful to present an algorithm for technique selection and propose a comprehensive classification. This was based on a single surgeon’s experience with 25 consecutive bipedicled free flap breast reconstructions. We predict our algorithm will optimize patient outcomes for this complex microvascular procedure.

## Patients and Methods

All patients undergoing unilateral bipedicled abdominal free flap breast reconstruction were identified from a prospective database of microsurgical procedures carried out at Cambridge University Hospital, UK. All procedures were performed over a 40-month period from November 2010 to March 2014 by a single surgeon (CMM).

Data collected included indications, microvascular anastomotic details and flap outcomes. In addition, patient demographics, breast size, previous adjuvant therapy, co-morbidities, inter-rib space type and width were recorded. Collation of the patient demographics, BMI, mastectomy weights, flap weights, inter-rib space distances, surgery duration and flap ischemia times was carried out using Microsoft Excel™ Software.

### Flap selection: bipedicled DIEP versus unipedicled DIEP

Patients requiring autologous tissue reconstruction based on their need for adjuvant radiotherapy or refusal to accept implant-based reconstructions were considered for abdominal tissue reconstruction. The decision to perform a bipedicled flap rather than a standard unipedicled flap was based on clinical assessment of the donor abdomen by the senior author (CMM). This was based on an estimate that three quarters of the lower abdominal tissue (which can be reliably perfused by one set of deep inferior epigastric vessels) would not be sufficient to reconstruct the required breast size. This took into account increasing the abdominal tissue available by schamfering the harvest while enabling safe closure of the donor site. An underlying assumption was (the nearly universally held view) that DIEP flaps are preferable to free TRAM flaps in view of the donor site morbidity. No intraoperative tests, such as clamping of the one set of perforators, were undertaken to determine the horizontal extent of the flap perfusion.

### Flap harvest

All patients were marked up the day before surgery. The perforators were located using an 8 MHz Doppler probe. Later in the series, all patients (*n* = 15) underwent CT angiography of the abdominal wall vessels (DIEP and SIEA). The flap was raised in a standard fashion from lateral to medial then inferior to superior and then superior to inferior carefully identifying and preserving the perforators. The SIEA pedicle was utilized if the artery was deemed suitable with good pulsatility and a diameter of >1 mm (3). The dissection end point of the SIEA pedicle was at the femoral vessel junction, beyond the artery bifurcation. This is important in ensuring a large enough artery and minimizing discrepancy with the recipient arteries.

For DIEP flaps, the perforators were exposed by carefully incising the rectus sheath with a size 15 blade without excising any of the sheath. The perforator course was then followed through the muscle up to the main pedicle carefully ligating the side branches. At least 2 cm of vessel was preserved at the superior continuity and inferior continuity of the DIE vascular pedicles in case these were needed for *intraflap* anastomoses.

The pedicle was dissected toward its origin from the external iliac vessels aiming beyond the confluence of the venae commitantes. This was repeated on the opposite side.

The side with the best perforators became the primary flap and anastomosed anterograde to the internal mammary vessels. In the entire series, one flap was divided into two hemiflaps and rotated 90° to lie vertically adjacent to each other in the breast pocket (in order to increase its width).

### Recipient vessel preparation

This utilized the total rib-preservation technique detailed in the author’s previous publication (15). The IMVs were exposed in the second intercostal space (ICS) without cartilage sacrifice but removal of the intercostal muscles. Occasionally, the third ICS was exposed in order to try and identify veins proximal to any confluence.

### Rectus sheath closure

When required, the opening(s) in the rectus sheath was closed using looped “0” nylon over-and-over continuous sutures in two layers. If the flap configuration utilized two DIE vascular pedicles repair of the two incisions in the sheaths was performed simultaneously by two surgeons to avoid unequal distribution of tension and facilitate closure of the second side. Mesh reinforcement was not used in any of the patients.

### Vessel selection for anastomoses

This is detailed in the algorithm (*vide infra*).

## Results

**Figure 1:**
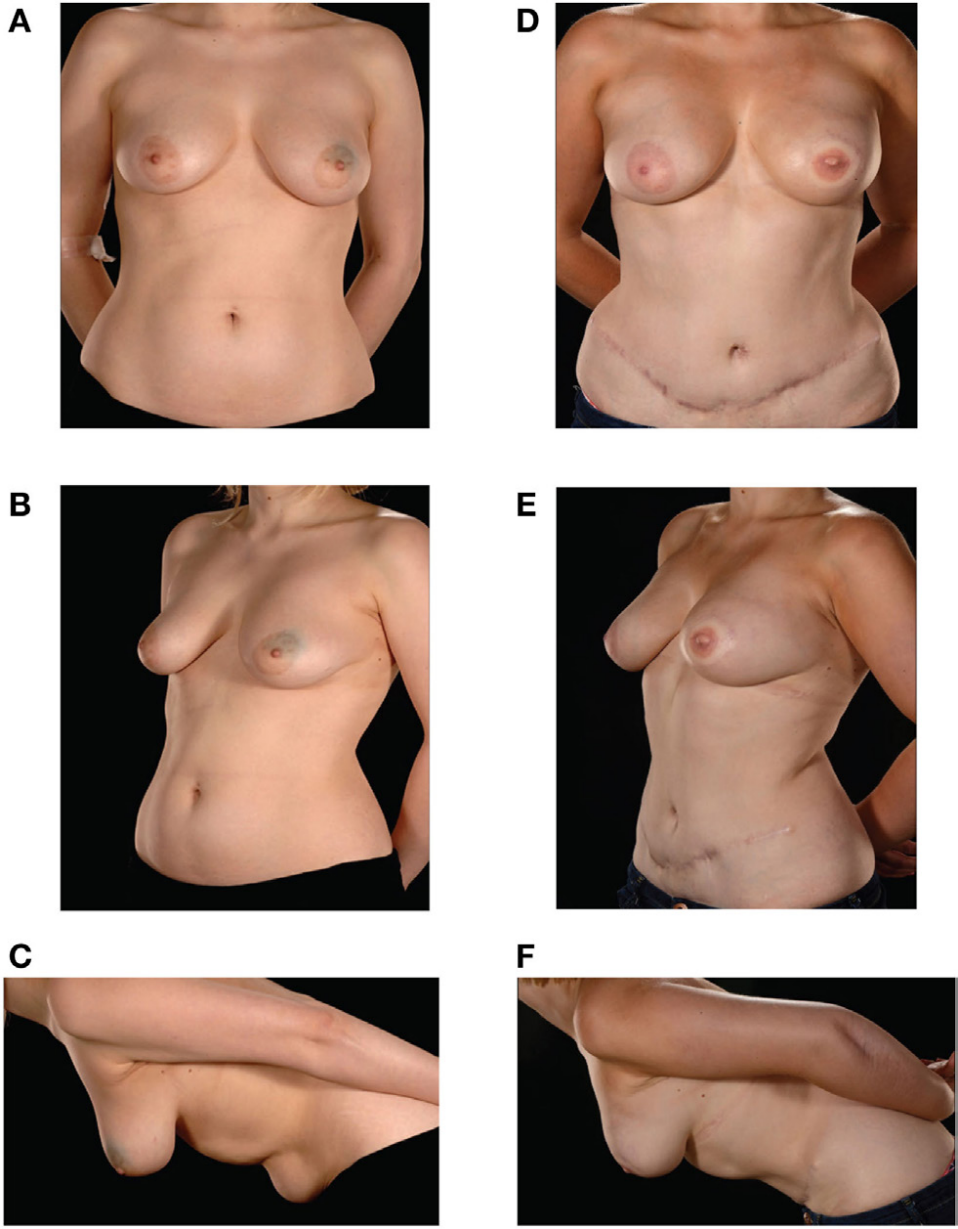
**Preoperative (A–C) and postoperative (D–F) appearances of a 26-year-old patient with size D cup breasts**. She received an extraflap configuration DIEP–DIEP bipedicled free flap breast reconstruction.

**Figure 2:**
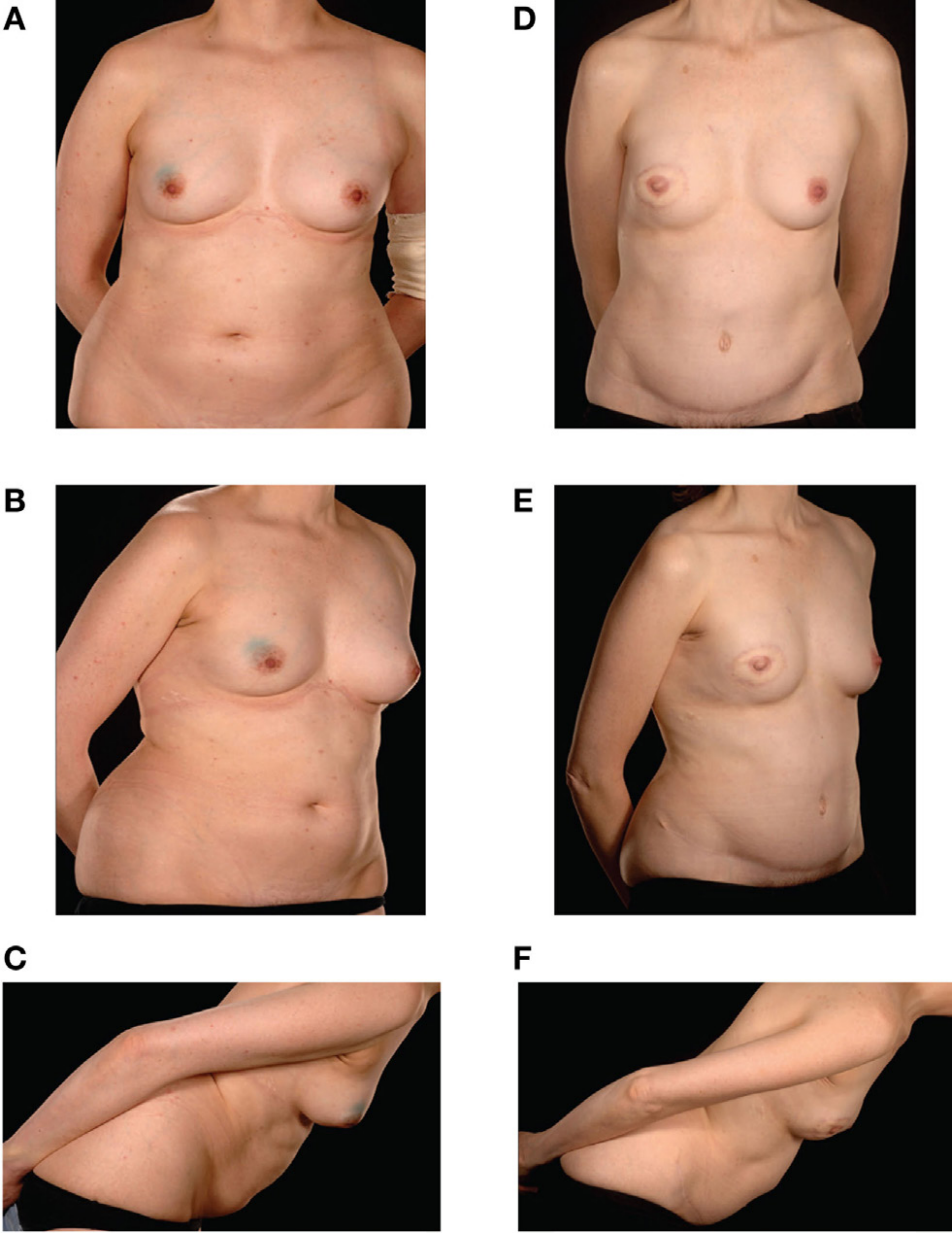
**Preoperative (A–C) and postoperative (D–F) appearances of a 43-year-old nulliparous patient with size B cup breasts who required postoperative radiotherapy and refused to countenance the idea of an implant-based reconstruction**. She underwent a Type IIIb DIEP–DIEP bipedicled flap reconstruction.

**Figure 3:**
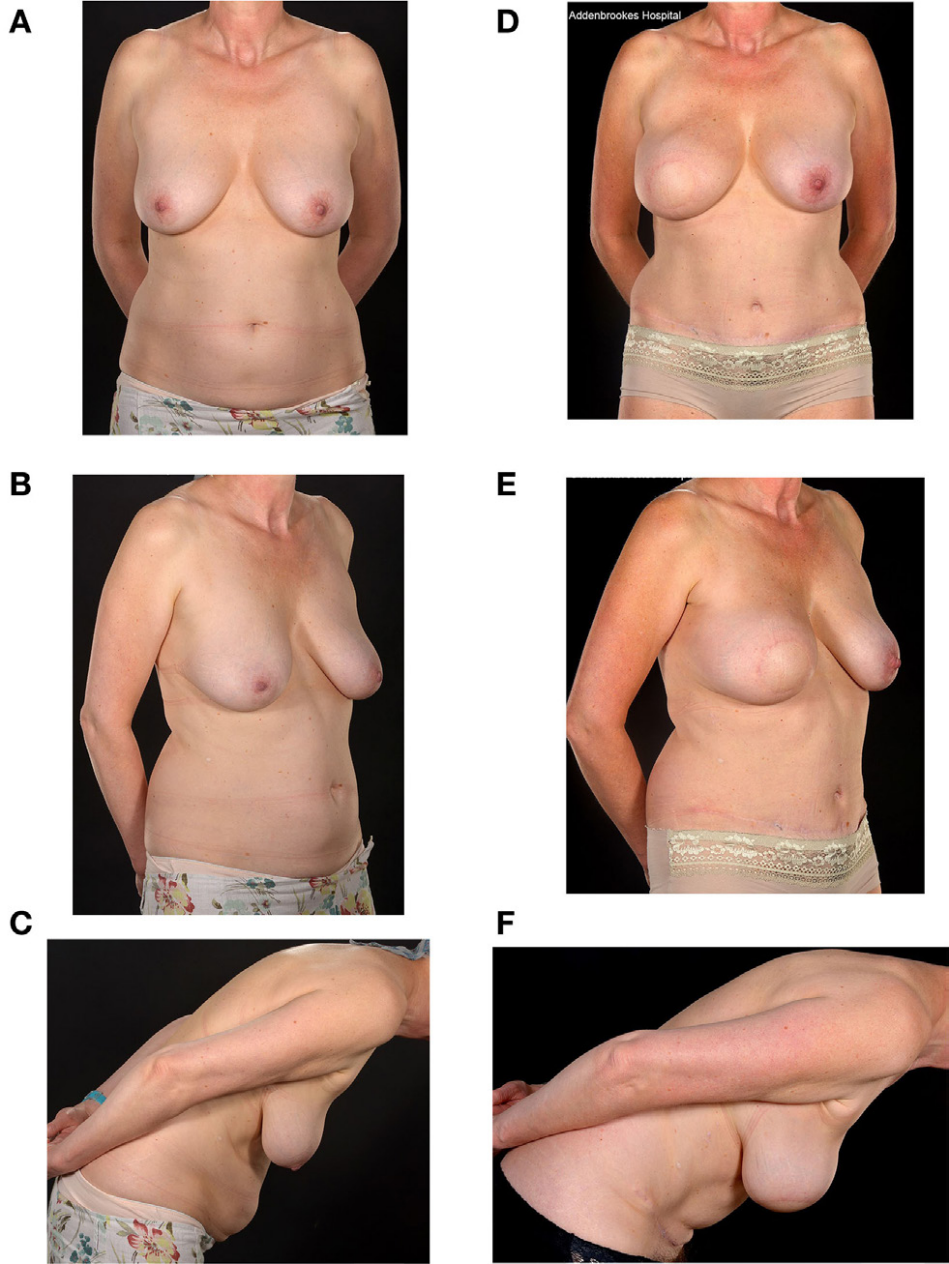
**Preoperative (A–C) and two-year postoperative (D–F) and post-radiation appearances of a 46-year-old nulliparous patient with large breasts (size E cup) and a relatively small abdomen**. She underwent a Type 1 SIEA-SIEA double-pedicled free flap breast reconstruction at the time of her therapeutic mastectomy. She has hitherto declined nipple reconstruction.

Over the 40-month period, a total of 112 free abdominal flaps were performed in 98 consecutive patients by one surgeon. 25 (22%) had a bipedicled vascular configuration (Table 2). The mean patient age was 45 years (27–54). The median BMI was 23.7 kg/m^2^ (20.2–28.7). The bra cup sizes were 2 As, 3 Bs, 9 Cs, 7 Ds, 2 Es, and 2 Gs. The indications for surgery were (alone or in combination): inadequate abdominal tissue for one breast, refusal of implant-based reconstruction, small breast volume or planned postoperative radiotherapy (Figures 1–3). Two patients had failed prosthetic reconstructions salvaged with bipedicled free flaps. No patients had midline abdominal scars.

**Table 2:** **Bipedicled abdominal free flap patient summary**.

Case no.	Age	BMI	Cup size	Immediate (I)/delayed (D)	Flap configuration	Ischemia duration (min)	Surgery duration (min)	Rib space width (mm)	Adjuvant postoperative radiotherapy	Complications
1	26	24.9	34B/C 32DD	I	DIEA/DIEA extraflap	104	770	14.5	No	
2	43	23.4	34B/C	I	III b	98	744	20	Yes	
3	38	23.1	32A/B	I	II b	83	780	24	Yes	Requested liposuction
4	45	26	36B	I	III a	91	690	19	Yes	
5	51	25.3	34C	I	DIEA/DIEA extraflap	90	600	26	No	
6	43	24	36B/C	Salvage	III a	110	790	21	No	
7	43	24.7	34D	I	DIEA/DIEA extraflap	65	600	26	No	
8	46	20.2	36C	I	II b	115	748	27	No	Requested liposuction
9	50	22.8	34B/C	I	DIEA/DIEA extraflap	81	716	15	No	
10	42	22.9	36D	I	II a	82	690	15	Yes	Small area fat necrosis
11	47	20.3	34C	I	III a	190	675	16	Yes	Requested liposuction
12	40	22.5	36B	I	DIEA/DIEA extraflap	76	654	19	Yes	Abdominal wound dehiscence
13	46	23.9	36B	I	DIEA/DIEA extraflap	125	650	23	Yes	
14	44	21.3	32D	I	III b	101	554	20	Yes	
15	54	23.9	32D	I	DIEA/DIEA extraflap	106	572	26	Yes	
16	53	23.4	34D	I	DIEA/DIEA extraflap	130	647	26	No	
17	53	25.2	32G	I	DIEP-DIEA extraflap	157	554	17	Yes	
18	46	23	32E	I	SIEA/SIEA extraflap	118	622	20	Yes	
19	34	18.7	34D	I	DIEP–DIEP extraflap	129	693	14	Yes	
20	48	28.8	34E	Delayed	II b	55	535	14	Yes. Pre-op	
21	50	26.2	32D	I	DIEA–DIEA extraflap	87	670	24	No	
22	45	24.3	36AA	Delayed	DIEA–DIEA extraflap	107	582	22	Yes	
23	48	25	32G	I	DIEA–DIEA extraflap	182	691	18	No	
24	49	23.5	36C	I	DIEP–SIEA extraflap	92	702	19	No	
25	26	26	36C	Salvage	DIEP–DIEP extraflap	60	468	22	Yes. Pre-op	

**Figure 4:**
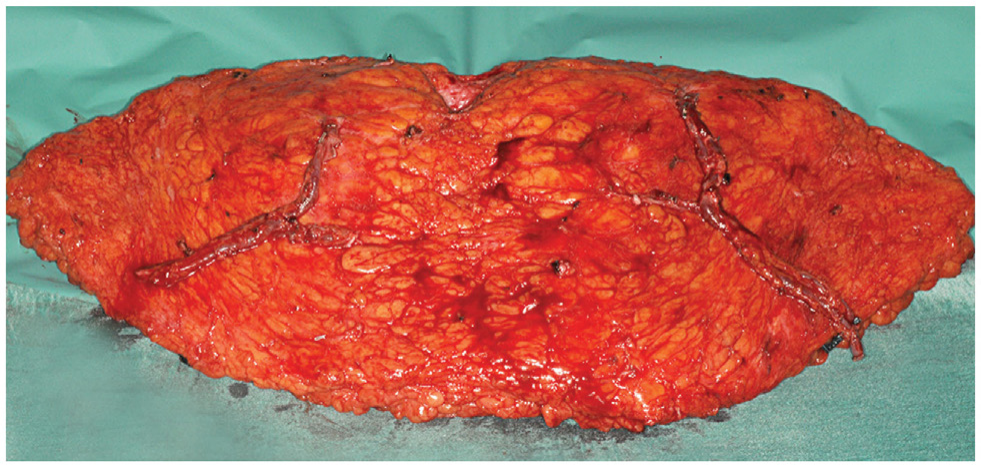
**An intraoperative photograph of a bipedicled DIEP–DIEP flap after harvest prior to micro-anastomoses**. Each hemiflap is based on two perforators.

**Figure 5:**
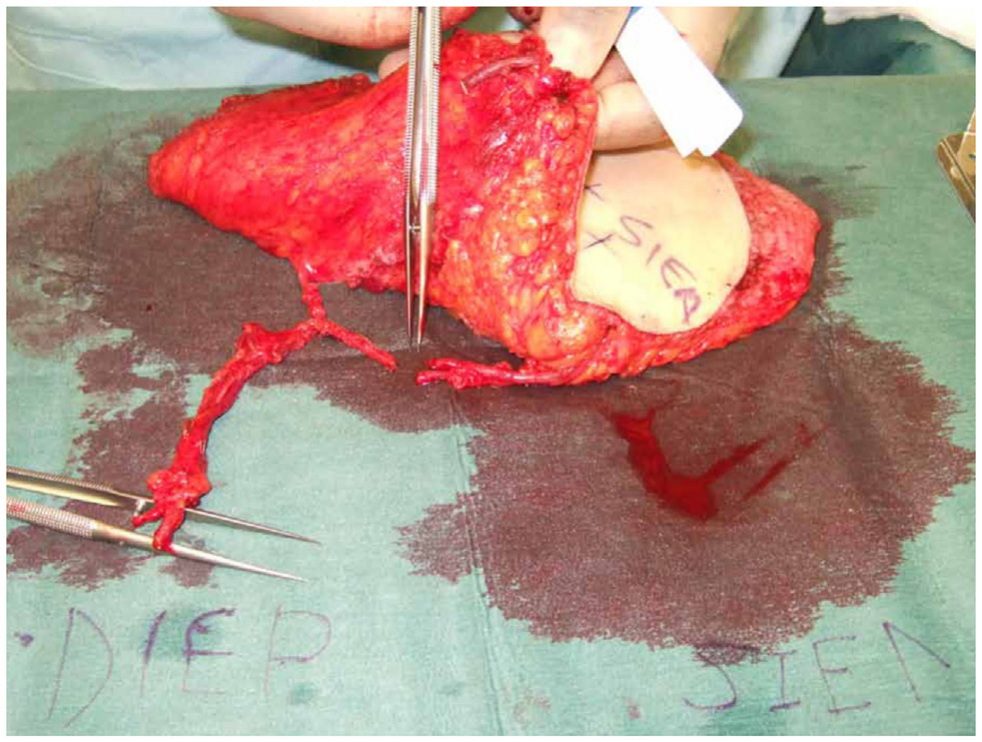
**An intraoperative photograph of a DIEP–SIEA showing the ease of anastomoses of the SIEA second pedicle to the superior continuity of the DIEP primary vascular pedicle**.

The median mastectomy weight was 445 g (range = 220–896) with a median flap weight of 618 g (range = 432–1275). The microvascular anastomoses used (Table 3) were DIEA/DIEA extraflap in 15 patients (Figures 1 and 4), DIEA/DIEA intraflap in four (two superior and two inferior “continuities”) (Figure 2), DIEA/SIEA extraflap in one, DIEA/SIEA intraflap in four (two superior and two inferior “continuities”) (Figure 5) and SIEA/SIEA in one patient (Figure 3).

**Table 3:** **Summary of the vessel constructs used in this bipedicled abdominal free flap series**.

Name of construct	DIEP/DIEP	DIEP/SIEA	SIEA/SIEA
Inferior continuity	2	2	–
Superior continuity	2	2	–
Extraflap anastomoses	15	1	1
Totals	19	5	1

The median surgery duration was 670 min (468–790) with flap ischemia time of 103 min (24–190). Twenty-one reconstructions were immediate, two were delayed and two were salvage (tertiary). All patients used the total ipsilateral rib-preservation method of IMV harvest. The second ICS was used in 17 patients and both second and third spaces in 8 patients. The widths for the ICSs ranged from 14 to 27 mm for the second space and 11 to 25 mm for the third.

The arterial anastomoses were all performed end-to-end using interrupted 9/0 monofilament nylon (S&T^®^). The venous anastomoses were undertaken with a venous coupler in 45 anastomoses (83%) and with 9/0 MFN continuous suture in eight (17%). The vein of the second flap was anastomosed to the bifurcated anterograde IMV vein in 6 cases (24%), its retrograde limb in 14 (56%), intraflap superior continuity 5 (20%), intraflap inferior continuity 3 (12%) and to the thoracodorsal vein in 1 (4%) (Table 4). The intercostal perforators and the pectoral vein were not used. The secondary pedicle arterial anastomoses were the retrograde IM artery in 16 cases (64%) with identical figures for the superior and inferior “continuities” and the TDVs. There were no anterograde internal mammary secondary pedicle arterial anastomoses [as these would have to be end-to-side (ETS) on the same IMA used as the recipient for the primary pedicle].

**Table 4:** **Summary of recipients used for venous and arterial anastomoses for the secondary flap**.

Recipient vessels	Venous number (%)	Arterial number (%)
Retrograde IM vessel	11 (44)	16 (64)
Anterograde IM vessel	6 (24)	0 (0)
Superior pedicle continuity	5 (20)	5 (20)
Inferior pedicle continuity	3 (12)	3 (12)
Thoracodorsal vessel	1 (4)	1 (4)
Intercostal perforator	0 (0)	0 (0)
Pectoral vein	0 (0)	0 (0)

All flap reconstructions were successful with no partial flap losses and no re-explorations. One patient needed on-table revision of the primary arterial anastomosis whilst another two required venous anastomoses revision due to coupler device malfunction. Where indicated (*n* = 14) the immediate reconstructions received adjuvant radiotherapy post-operatively without delay. One patient developed a 2-cm area of fat necrosis laterally on the SIEA flap side and this was managed conservatively. Another patient experienced exacerbation of a pre-existing lower abdominal bulge currently being treated conservatively. She also had minor dehiscence of her abdominal incision possibly due to smoking history. There have been no complaints of herniation or symptomatic abdominal wall tightness after a median follow up of 18.5 months (1–40). Three patients requested liposuction to reshape their reconstructed breasts.

## Discussion

Bipedicled free abdominal flaps widen the pool of patients who can benefit from the advantages of autologous tissue breast reconstruction. This series shows that the technique of transferring the entire lower abdominal flap on two vascular pedicles is reliable whilst being associated with minimal morbidity. The indications for double-pedicled flaps in the present series were usually a combination of factors which included the patient’s body habitus (relative size of the breast versus the lower abdomen) and their preference for totally autologous reconstruction. This was especially when adjuvant radiotherapy was planned. The decision whether to undertake a unipedicled or bipedicled DIEP flap was a clinical one made preoperatively in the ­outpatient clinic.

Previous published series have discussed various options for the arrangement of transferred tissue and microvascular configurations (Table 1). There are four described flap arrangements once the abdominal tissue is transferred onto the chest wall (5–10, 14–17). In the “*stacked*” variety the flap is split into two and positioned one on top of the other. The “*coned*” variant contours the horizontally orientated flap to various degrees. In a third option the bipedicled flap is rotated 90° to be vertical and “*folded*” over. This is useful for narrow breasts. The fourth type splits the flap into two, rotates each hemiflap 90° toward each other then arranges them vertically “*adjacent*” to each other. These arrangements are designed to maximize volume but must not be viewed in isolation from the microvascular anastomotic permutations.

**Figure 6:**
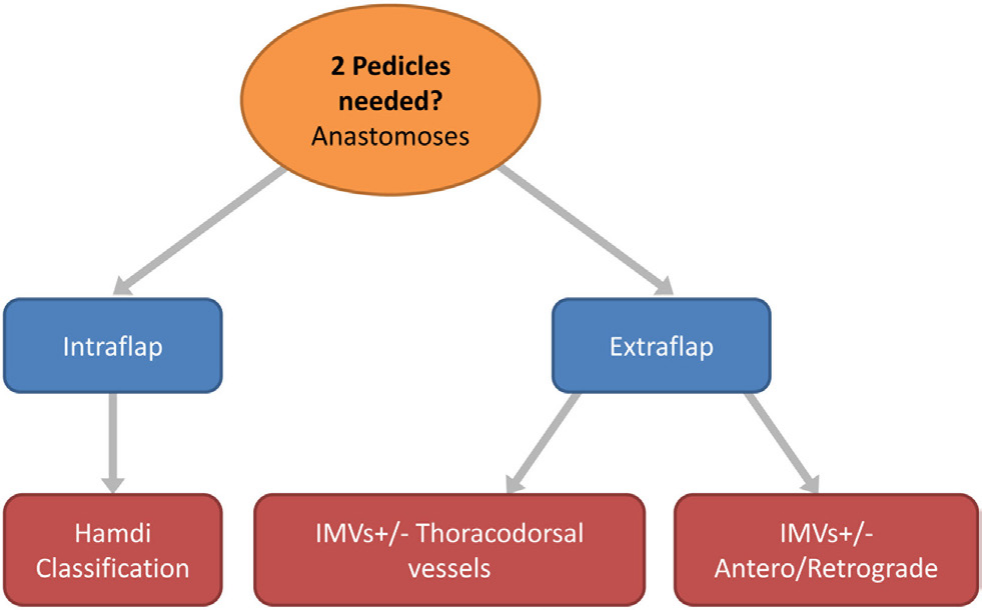
**Flow chart depicting the options in free flap vascular pedicle design for bipedicled microvascular flap anastomoses**.

**Figure 7:**
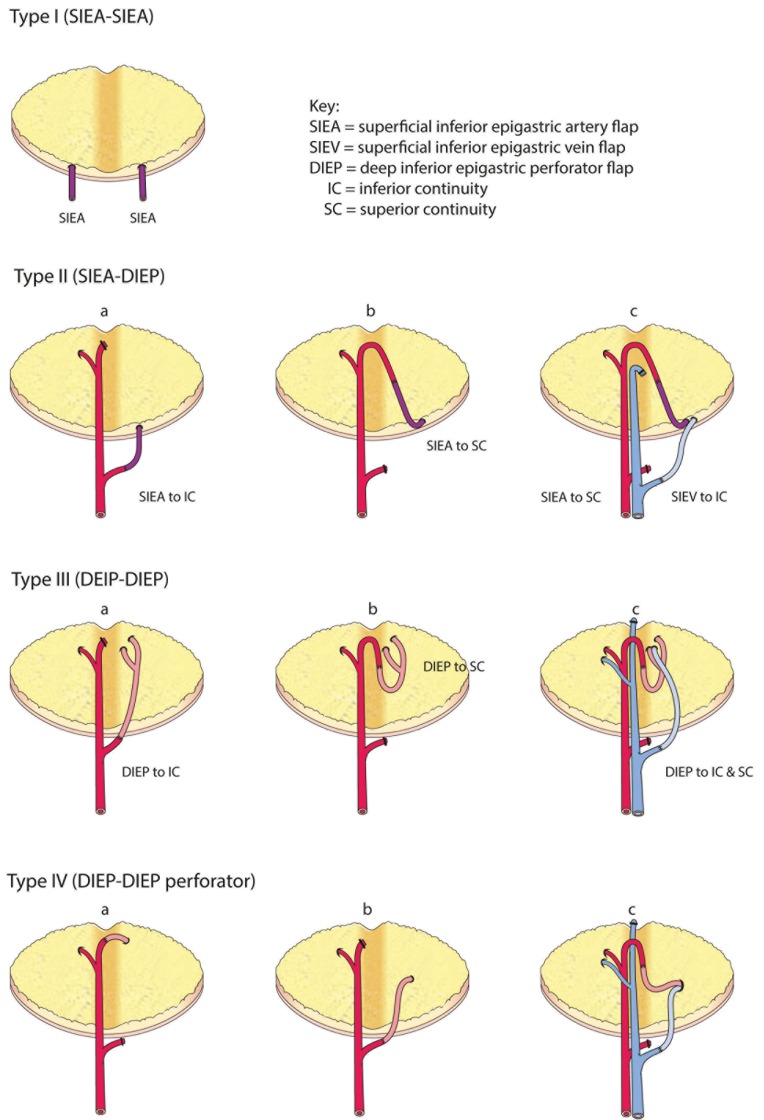
**Comprehensive classification of the variations for intraflap anastomoses in bipedicled abdominal free flaps (modified after Hamdi et al.)**.

**Figure 8:**
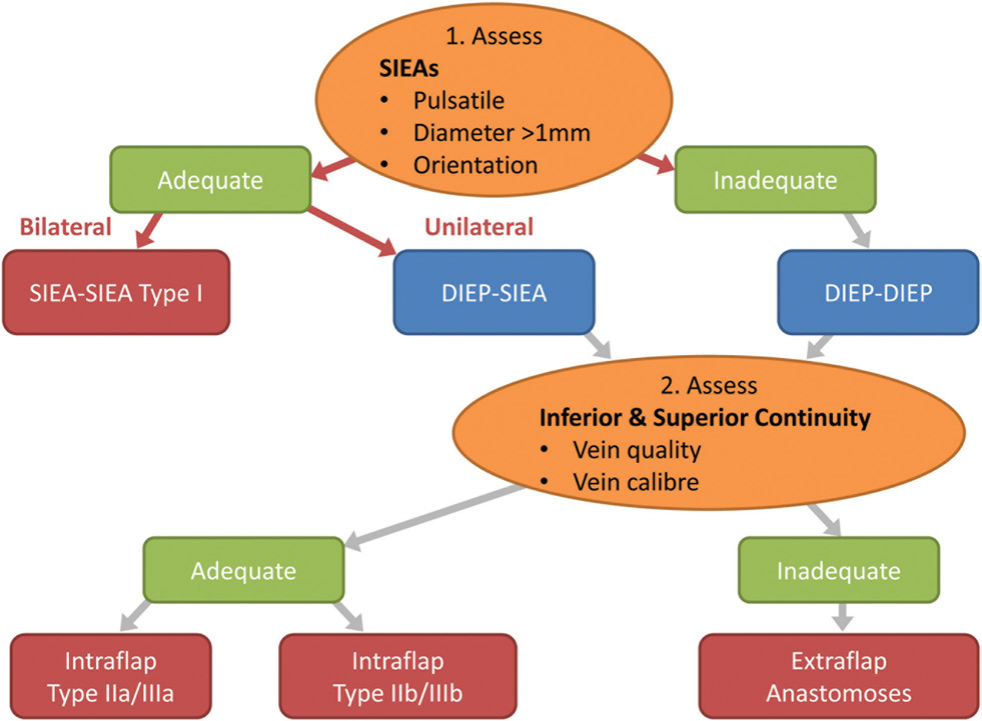
**Flow chart depicting the steps in decision making for the configuration of the flap vessels in abdominal double-pedicled free flap microvascular anastomoses**.

**Figure 9:**
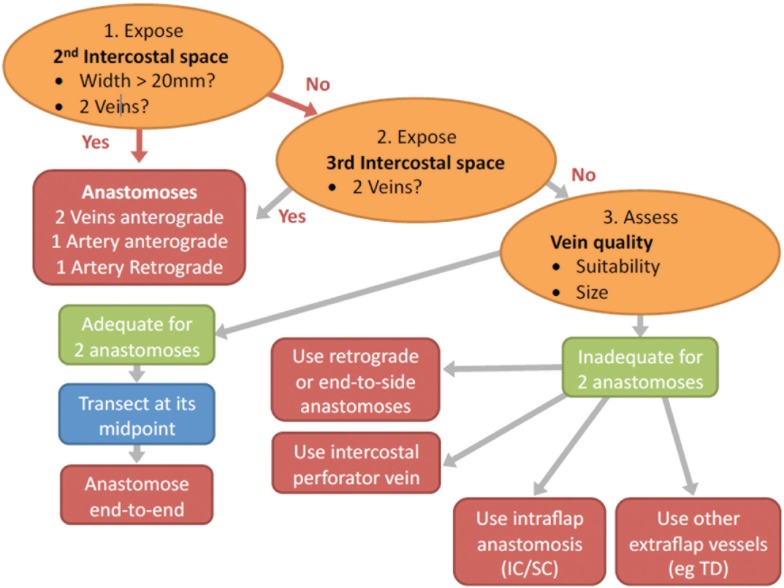
**Flow chart depicting the steps in decision making for determining the configuration of the recipient vessels in abdominal double-pedicled free flap microvascular anastomoses**.

The anastomoses for the second vascular pedicle can be performed *intraflap*, *extraflap*, or rarely both *intraflap*–*extraflap* (Table 4; Figure 6). The *intraflap* configurations consist of anastomosing the secondary vascular pedicle to branches of the primary flap’s vascular pedicle as detailed by Hamdi et al. (Figure 7). In the *extraflap* arrangement the secondary flap’s vessels are separately anastomosed retrogradely to the internal mammary vessels or anterogradely to the TDVs or other chest wall vessels (5–10, 14–17) (Table 1). Although all these articles report good outcomes, there is a wide variability in the microvascular configurations used. From our experience we have identified two points in the procedure when careful consideration of the arrangement to use for the microvascular anastomoses is critical. The first is the selection of pedicle design during flap harvest (Figure 8), and the second is the combination of recipient vessels for anastomoses (Figure 9), especially when an *extraflap* configuration for two pedicles is required. We have found that preoperative CT angiography can assist our vessel selection.

Intra-operatively we therefore make the following considerations. The superficial inferior epigastric (SIE) vessels are evaluated initially for pulsatility, size and location (Figure 8). This pedicle should be explored first because it is easier and quicker to dissect and gives less donor site morbidity. The SIEA vascular pedicle may provide good flap perfusion whilst avoiding trauma to the rectus abdominis muscle and fascia, such as that required for the raising of a DIE pedicle (3). However, occasionally the SIE vessels may have been injured in previous abdominal surgery, so care needs to be taken over their selection. If both arteries have a good caliber of >1 mm and display good pulsatility and orientation, then they are determined to be adequate and a bilateral SIEA Hamdi type I flap can be fashioned. If not, then the DIEAs must be considered next.

In our experience the easier and faster-to-dissect lateral row perforators can be used preferentially as an adequate basis of the bipedicled DIEP flap as there is sufficient perfusion of Hartrampf zones IV or III across the flap midline. This is in contrast to the situation in a unipedicled DIEP free flap where the more centrally located medial row perforators are to be preferred. Usually the medial row perforators are larger than lateral row ones and single (medial row) perforator unilateral DIEP pedicles are commonplace. When based on the lateral row we prefer to use at least two perforators in unipedicled flaps to be assured of overall flap perfusion and especially venous drainage. However, if two DIEA pedicles are being used then one lateral row perforator on each side should be adequate.

The next important stage is the evaluation of the superior and inferior “continuities” and branches of the DIE system (Figure 8; step 2). We have found that the superior “continuity” of the pedicle can be fragile, especially the venae comitantes, and should be assessed before the pedicle is divided as its vessels collapse post-division and become difficult to evaluate. The critical consideration here is the vein, which tends to be more friable due to its thinner wall. If both the artery and vein of the primary pedicle superior “continuation” have a sufficient caliber and quality, then a superior “continuity” intraflap anastomosis can be made with the secondary pedicle (Hamdi Type IIb or IIIb) (Figures 5 and 6). If not, the inferior continuity is considered, which usually supplies the medial row perforators. When basing the flap on lateral row perforators, the ligation of the division leading up to the medial perforators must leave an adequate “stump” for use in Hamdi type IIa and IIIa flap variations (6, 14). Rarely, an artery from the superior “continuity” can be used with a vein from the inferior “continuity” (14, 15) or vice versa. If these “continuities” are not suitable then the second pedicle must be anastomosed in an extraflap arrangement. We avoid the ETS intra-flap anastomoses described by Xu et al. because of the vagaries of this anastomotic type and the concern that it might thrombose due to turbulence at the anastomosis as a consequence of its close proximity to the primary flap anastomosis.

For the recipient vessel selection (Figure 9) all the patients in our series had their IMVs exposed using the senior surgeon’s total rib-preservation technique (18). In the entire series the primary flap artery was anastomosed to the IMA in an anterograde fashion. The secondary flap artery was anastomosed to the retrograde IMA limb in 64% (16/25) of cases. This reflects the practical ease of a single recipient site and the well-documented adequate retrograde arterial flow (19, 20).

Selecting the appropriate vein for the second pedicle is another crucial step in ensuring pedicled free flap success. Using the same IMV recipients for both pedicles is beneficial because only one recipient site needs to be exposed and the position facilitates the flap inset and shaping. Ideally, two anterograde veins would be used as the recipient vessels to avoid any valves in the retrograde veins (21). We preferentially use the second ICS because of its numerous advantages (18). If the second space is small (<20 mm) we sometimes also expose the third space (Figure 9). This serves two purposes:

(a) It may expose the IMV venous confluence and thus provide the easier anterograde venous anastomoses. Often the vein confluence is just under the cranial edge of the third costal cartilage (22, 23).
(b) It lengthens the limbs of the vessels to be used for anterograde and retrograde anastomoses.

However, care must be taken when dissecting the vessels under the third rib cartilage. This is aided by the observation that there are no side branches of the IMVs directly under the cartilage where visibility would be difficult. The need for meticulous ligation of any tributaries at the superior and inferior aspects of the third costal cartilage is obvious. To facilitate the anastomoses, the third costal cartilage could be sacrificed as in the traditional method of IMV exposure, but we have not found this to be necessary in our series.

**Figure 10:**
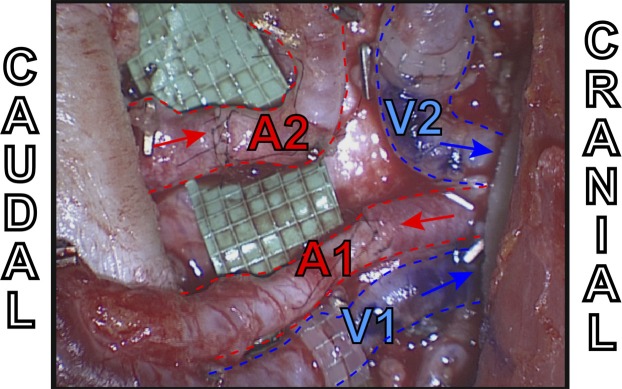
**Intraoperative example of extraflap DIEA–DIEA anastomoses to the internal mammary vessels using two anterograde veins (V1 and V2) both with venous couplers, one antegrade artery (A1) and one retrograde artery (A2)**.

If two anterograde IM veins are not available we resort to the use of the retrograde limb. In this procedure the vein is clamped with 2 V (venous) Acland clamps at its caudal and cranial ends in the second space and divided at its midpoint (Figure 9; step 3). The veins of the primary flap and secondary flaps are anastomosed anterograde and retrograde respectively using venous couplers (Figure 10). Some authors have advocated flushing the retrograde vein lumen with heparinized saline to identify any valves or resistance caused by obstruction. We do not believe this to be of any to be of practical significance. If the retrograde limb is not suitable then we consider an *intraflap* vascular arrangement by anastomosing the vein to the superior or inferior continuity.

There may be the rare occurrence in which the veins in both the second and third ICSs are inadequate. In this eventuality ETS anastomosis of the veins may be considered, although none of our cases required this option.

We would only use the TDVs in the axilla as a last resort. Their use would compromise any subsequent use of a salvage latissimus dorsi flap, make the flap inset difficult with anastomoses in two different anatomic locations and lead to lengthened surgical time due to second recipient vessel site exposure. Combined usage of the IMVs and the TDVs for bipedicled anastomoses, however, becomes useful in chest wall reconstruction as the “flattened” flap adequately covers a wide chest defect. It is also easier to use the TDVs if the reconstruction is immediate with an associated axillary lymph node clearance. This exposes the vessels in advance, a key advantage of the subscapular-thoracodorsal system (24, 25).

To ensure good perfusion of the main flap, we perform the primary pedicle anastomosis first after exposing and evaluating the recipient vessels. This also reduces the risk of thrombosis at the secondary anastomoses. In contrast, Xu et al. and the Broomfield group carry out the *intraflap* anastomosis initially (7, 10). We prefer using a coupler for all our venous anastomoses as it is quick, technically easier and helps reduce flap ischemia time. After full flap perfusion, the flap can be inset in a *coned*, *folded*, *stacked*, or *adjacent* arrangement taking great care not to disrupt the anastomoses. When folding the flap we place the flexure inferiorly in order to give the best esthetic appearance, but it can be made superiorly depending on the breast projection desired.

The disadvantages of bipedicled free flaps are their technical complexity, difficulty in flap inset and shaping, tightness of the abdominal closure, prolonged surgical duration and cost. However, we believe that by considering the crucial factors discussed above the surgeon can minimize the donor site morbidity, reduce the duration of flap harvest and vessel anastomosis and optimize the ease of flap inset. From our experience we have proposed a treatment algorithm in Figures 8 and 9 and a comprehensive classification of bipedicled free flap abdominal reconstructions (Table 5; Figure 7).

**Table 5:** **The Malata–Rabey comprehensive classification of double pedicled abdominal free flap anastomoses**.

A. Intraflap anastomoses
**Type I** SIEA – SIEA
**Type II** SIEA – DIEP
a. Inferior continuity
b. *Superior continuity*
c. *Combination inferior and superior (vein and artery)*
**Type III** DIEP – DIEP
a. Inferior continuity
b. Superior continuity
c. *Combination of inferior and superior (vein and artery)*
**Type IV** DIEP – DIEP Perforator (left or right)
a. *Inferior continuity (unlikely unless centrally located and long IC)*
b. *Superior continuity*
c. *Combination of inferior and superior (artery and vein): highly unlikely*
**Type V** DIEP – DIEP
a. *End-to-side anastomosis (artery alone) (vein to vena comitans)*
b. *End-to-side anastomosis (artery and vein)*
B. Extra-flap anastomoses
**Type I** To a single recipient site: IMVs
a. Flap 1 (both vessels antegrade); Flap 2 (artery retrograde, vein antegrade to second vc)
b. Flap 1 (both vessels antegrade); Flap 2 (both artery and vein retrograde)
**Type II** To a single recipient site: thoracodorsal system
a. Flap 1 (both vessels antegrade); Flap 2 (artery retrograde, vein antegrade to vc)
b. Flap 1 (both vessels antegrade); Flap 2 (both retrograde) – unlikely
**Type III** To two separate recipient sites
a. IMVs and subscapular-thoracodorsal system (preferably above serratus branch)
b. IMVs and pectoral vessels (unlikely)
c. IMVs and other vessels (cephalic vein loop, etc) – when in “trouble”
C. Combined intraflap and extra-flap anastomoses

## Conclusion

Bipedicled free flaps can sometimes be necessary in patients undergoing abdominal flap breast reconstruction and can be successfully undertaken in large microvascular surgery centers. They are a reliable and safe option for unilateral autologous breast reconstruction when the volume of tissue required to make a breast mound is larger than that can be transferred on a single flap pedicle. They can greatly benefit the correct patient.

There are numerous techniques for the microsurgical constructs of bipedicled flaps and surgeons intending to perform this surgery should be familiar with them. Our series shows that double-pedicled abdominal free flaps can be safely undertaken with total rib-preservation technique of internal mammary vessel exposure. The algorithm that we have derived from our experience can assist surgeons embarking on this complex microsurgery if they adopt the discussed principles.

## Conflict of Interest Statement

The authors declare that the research was conducted in the absence of any commercial or financial relationships that could be construed as a potential conflict of interest.
